# Normally Off AlGaN/GaN MIS-HEMTs with Self-Aligned p-GaN Gate and Non-Annealed Ohmic Contacts via Gate-First Fabrication

**DOI:** 10.3390/mi16040473

**Published:** 2025-04-16

**Authors:** Yinmiao Yin, Qian Fan, Xianfeng Ni, Chao Guo, Xing Gu

**Affiliations:** Institute of Next Generation Semiconductor Materials, Southeast University, Suzhou 215123, China; 220224568@seu.edu.cn (Y.Y.); 103200035@seu.edu.cn (Q.F.); 103200036@seu.edu.cn (X.N.); 101010759@seu.edu.cn (C.G.)

**Keywords:** AlGaN/GaN HEMT, enhancement-mode, gate-first process, metal-insulator-semiconductor (MIS), non-annealed ohmic contacts

## Abstract

This study introduces an enhancement-mode AlGaN/GaN metal-insulator-semiconductor high-electron-mobility transistor (MIS-HEMT) featuring a self-aligned p-GaN gate structure, fabricated using a gate-first process. The key innovation of this work lies in simplifying the fabrication process by utilizing gate metallization for both electrical contact and etching mask functions, enabling precise self-alignment. A highly selective Cl_2_/N_2_/O_2_ inductively coupled plasma (ICP) etching process was optimized to etch the p-GaN layer in the access regions, with a selectivity ratio of 33:1 and minimal damage to the AlGaN barrier. Additionally, a novel, non-annealed ohmic contact formation technique was developed, leveraging ICP etching to create nitrogen vacancies that facilitate contact formation without requiring thermal annealing. This technique streamlines the process by combining ohmic contact formation and mesa isolation into a single lithographic step. Incorporating a SiNx gate dielectric layer led to a 4.5 V threshold voltage shift in the fabricated devices. The resulting devices exhibited improved electrical performance, including a wide gate voltage swing (>10 V), a high on/off current ratio (~10^7^), and clear pinch-off characteristics. These results demonstrate the effectiveness of the proposed fabrication approach, offering significant improvements in process efficiency and manufacturability.

## 1. Introduction

AlGaN/GaN high-electron-mobility transistors (HEMTs) leverage high-density two-dimensional electron gas (2DEG) channels, combining wide bandgap energy with low on-resistance and high breakdown voltage. Their intrinsically low gate charge facilitates high switching frequencies, making them promising candidates for power electronic applications [1]. However, the polarization-induced 2DEG formation at zero gate bias results in normally on operation for most HEMTs, posing safety risks, reliability challenges, and design complexity. Various strategies, including recessed gate architectures (thin-barrier heterostructures) [2,3], p-type cap layers [4,5], and fluorine ion implantation, have been explored to achieve enhancement-mode operation [6,7]. However, fluorine-based treatments demonstrate thermal instability, whereas recessed gates are prone to increased gate leakage currents. Although commercially available p-GaN gate HEMTs on Si substrates represent the current mainstream solution for enhancement-mode devices, their fabrication remains challenging due to complex processing requirements. In these structures, a thick p-GaN layer is typically grown on a thin AlGaN barrier, followed by gate metal deposition on the p-GaN cap. The excess p-GaN outside the gate region is then removed via dry etching. Self-aligned etching with the gate metal as an etch mask effectively reduces alignment errors while ensuring uniform potential distribution within the p-GaN layer. However, precise etch control combined with minimization of the AlGaN barrier damage is critical to maintaining high electron density in the access regions.

Conventional high-temperature (HT) annealing for ohmic contact formation risks gate degradation, as demonstrated by microcrystallites in gate dielectrics and elevated interface trap density after annealing, both of which exacerbate leakage currents [8,9]. To overcome this limitation, a non-annealed ohmic contact formation technique was developed using inductively coupled plasma (ICP) etching. This process creates nitrogen vacancies through controlled plasma-induced damage, which act as donor-like defects to establish ohmic conduction while preventing gate degradation [10,11]. Concurrently, the metallization achieves enhanced sidewall contact with the two 2DEGs, facilitating ohmic contact formation along the etched trench sidewalls [12]. Furthermore, the intrinsically low hole concentration in p-GaN confines threshold voltages ($V_{th}$) to sub-3 V ranges, which are inadequate for most practical applications. Moreover, excessive gate leakage currents severely limit the operational gate voltage window [13]. The integration of a dielectric interlayer between the gate metal and p-GaN has been demonstrated as an effective approach to enhance $V_{th}$ while suppressing leakage currents [14].

Herein, we present a novel normally off MIS-HEMT device integrating gate self-alignment technology with a gate dielectric layer, which fulfills the aforementioned requirements while reducing process complexity. The optimized etching process demonstrates an ICP etching selectivity of 33:1 for P-GaN over AlGaN through process parameter optimization, accompanied by ultralow etching-induced damage. Notably, the developed non-annealed ohmic process simultaneously implements ICP etching of ohmic contacts and mesa isolation—the first reported implementation for P-GaN gate devices to our knowledge—achieving ohmic characteristics while significantly simplifying process flow. The fabricated device exhibits a threshold voltage ($V_{th}$) of 4.5 V, positioning it at the forefront of reported values for comparable structures. Compared with conventional HEMT devices without gate dielectric layers, this architecture demonstrates a significant positive shift of +2.5 V in $V_{th}$. Moreover, the proposed device achieves an excellent electrical performance including a wide gate swing of 10 V, an on/off ratio exceeding $10^{7}$, while maintaining subthreshold swing and dynamic on-resistance (Ron) comparable to conventional dielectric-free HEMT devices.

## 2. Device Structure and Fabrication

**Figure 1:**
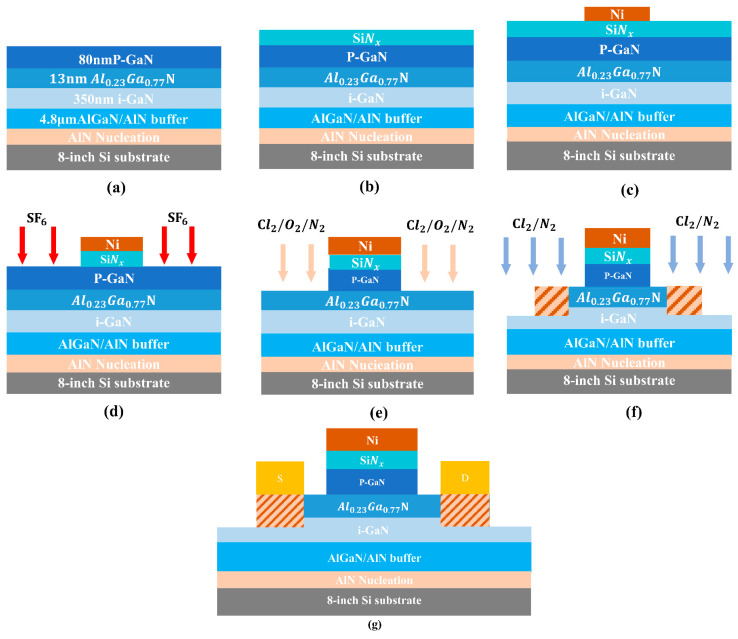
Schematic of device fabrication process flow. (**a**) Epitaxial structure formation. (**b**) Deposition of SiNx via LPCVD. (**c**) Deposition of gate metal Ni. (**d**) Etching of SiNx layer. (**e**) Selective etching of P-GaN layer. (**f**) Mesa isolation with simultaneous ohmic region etching. (**g**) Deposition of ohmic metal stack Ti/Al/Ni/Au with no subsequent annealing process, deviating from conventional ohmic contact formation protocols.

The E-mode AlGaN/GaN heterostructure was epitaxially deposited on 8-inch silicon substrates using a planetary Aixtron G5+ metal–organic chemical vapor deposition (MOCVD) system at Suzhou Hanhua Semiconductor Ltd. (Suzhou, China). Trimethylgallium (TMGa), trimethylaluminum (TMAl), and NH_3_ served as the gallium, aluminum, and nitrogen precursors, respectively. Prior to growth, the substrate underwent thermal annealing at 1100 °C in a hydrogen atmosphere to eliminate surface oxides. This was followed by an aluminum pre-deposition step conducted without NH_3_. The E-mode HEMT layers were subsequently grown, with the epitaxial structure (Figure 1a) consisting of the following sequential layers: an AlN nucleation layer, a 4.8-μm AlN/AlGaN superlattice buffer, a 350 nm GaN channel, a 13 nm Al_0.23_Ga_0.77_N barrier, and an 80 nm Mg-doped p-GaN cap layer designed for normally off operation. The p-GaN layer was doped with magnesium at a concentration of 2 × 10^19^ cm^−3^, with in situ activation achieved under a nitrogen ambient during MOCVD growth. The wafers were then fabricated into devices, and the schematic of the device fabrication process flow is depicted in Figure 1.

**Figure 2:**
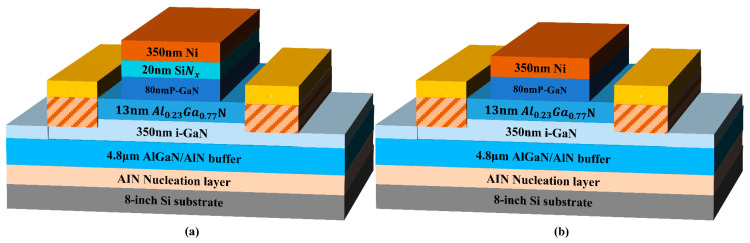
Schematic diagrams of (**a**) the insulator gate device structure and (**b**) the metal gate device structure.

The device fabrication is initiated with gate stack formation. Before processing, the as-grown wafer was subjected to sequential surface preparation: (1) oxide removal using buffered oxide etch (BOE, HF: H_2_O = 1:6) for 90 s, (2) ultrasonic cleaning in acetone and isopropanol each for 10 min. A 20 nm ${SiN}_{x}$ dielectric was deposited on the p-GaN surface by low-pressure chemical vapor deposition (LPCVD) at 785 °C and 300 mTorr, using N_2_ as carrier gas with dichlorosilane (DCS) and NH_3_ as precursors. The gate pattern was defined by photolithography, followed by electron-beam evaporation of a 350 nm-thick Ni layer and lift-off patterning. The Ni gate structure also served as a hard mask for subsequent p-GaN dry etching. A fluorine-based inductively coupled plasma (ICP) dry etch was first employed to remove the ${SiN}_{x}$ in non-gate regions. Subsequently, a low-damage, high-selectivity Cl_2_/O_2_/N_2_ (30/10/10 sccm) inductively coupled plasma (ICP) etching process was implemented with 100/25 W ICP/bias power and 15 mTorr chamber pressure to etch the p-GaN layer. The etching process was conducted for 5 min, including an over-etching duration of 1 min to ensure the complete removal of the p-GaN material, resulting in an etch depth of 80 nm. Benefiting from the high selectivity characteristics of this process, the etching was automatically terminated at the AlGaN interface. Post-etch damage was chemically passivated through immersion in 25 wt.% tetramethylammonium hydroxide (TMAH) solution maintained at 85 °C for 15 min. Mesa isolation with 120 nm depth was formed using Cl_2_/N_2_ ICP etching. Ohmic regions were defined through ICP etching, whereby plasma-induced nitrogen vacancies facilitated ohmic contact formation through donor-type defects, eliminating the requirement for high-temperature annealing and thus avoiding gate degradation. A Ti/Al/Ni/Au (20/130/50/50 nm) ohmic-contact metallization stack was deposited using electron-beam evaporation and defined by lift-off patterning. Additionally, a Ni metal–gate reference device (without dielectric) was fabricated for comparative analysis, The device with a gate dielectric layer will be referred to as the insulator gate device, and will be described using this terminology in the following text. The structural diagrams of the two types of devices are shown in Figure 2.

**Figure 3:**
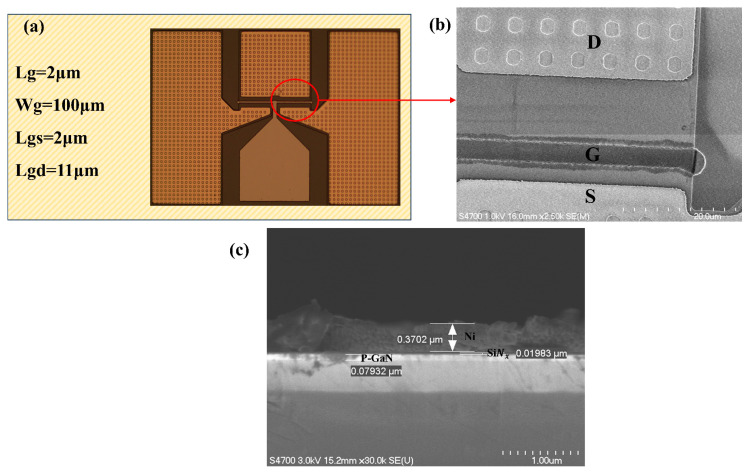
(**a**) Optical micrographs of the fabricated devices. (**b**) Top-view SEM image of the device structure, showing the regions labeled as D (Drain), G (Gate), and S (Source). The image was acquired using an accelerating voltage of 1.0 KV with a beam current of 10 μA. (**c**) Gate cross-section SEM image of a cleaved sample. Imaging parameters: 3.0 kV accelerating voltage and 10 μA beam current.

Figure 3a presents optical microscopy images of the fabricated devices, while Figure 3b shows a top-view SEM image of the fabricated device, with the gate, source, and drain electrodes clearly identified. Notably, the ICP-etched recess features of the ohmic contacts at the source and drain demonstrate distinct morphological properties. Figure 3c presents a cross-sectional scanning electron microscopy (SEM) image showing the Ni gate electrode, ${SiN}_{x}$ gate dielectric, and p-GaN layer.

## 3. Results and Discussion

### 3.1. Self-Terminated Etching of GaN over AlGaN

In AlGaN/GaN HEMTs, achieving an enhancement-mode (E-mode) operation requires complete depletion of the 2DEG under zero gate bias, implying that the AlGaN barrier must be sufficiently thin. However, the thin AlGaN barrier layer renders the 2DEG sheet carrier density ($n_{s}$) highly sensitive to both the unintentional recess depth formed in AlGaN during p-GaN etching and the presence of residual p-GaN material, as demonstrated in [15]. These dependencies significantly impact device output characteristics, underscoring the need for precise control of p-GaN etch depth while minimizing AlGaN surface damage.

Achieving a high etch selectivity between p-GaN and AlGaN—i.e., maximizing the p-GaN etch rate while minimizing (or nearly eliminating) the AlGaN etch rate—is critical to completely removing the p-GaN layer without damaging the AlGaN barrier. Chlorine-based dry etching demonstrates monotonic etch rate reduction correlating with elevated aluminum composition AlGaN [16]. Controlled oxygen introduction during plasma processing facilitates self-limiting (Al,Ga)O_x_ passivation layer formation via plasma-enhanced oxidation, effectively terminating the AlGaN etching process [17]. Tuning the competition between oxidative passivation and ion-assisted oxide removal enables active selectivity control throughout the etching process. The ICP etch rate and selectivity depend strongly on RF power, ICP power, and chamber pressure parameters. Through design-of-experiments on HanHua Semiconductor’s ICP system (Suzhou, China), we established parameter optimization protocols guided by established plasma-etch models [18,19], yielding high-selectivity, low-damage p-GaN etching over AlGaN. Specifically, reduced ICP/bias power (100/25 W) lowered the etch rate, while increasing chamber pressure to 15 mTorr shortened the ion mean free path, inhibiting etch product desorption. Oxygen flow rates were varied (0–10 sccm) to investigate its role.

**Figure 4:**
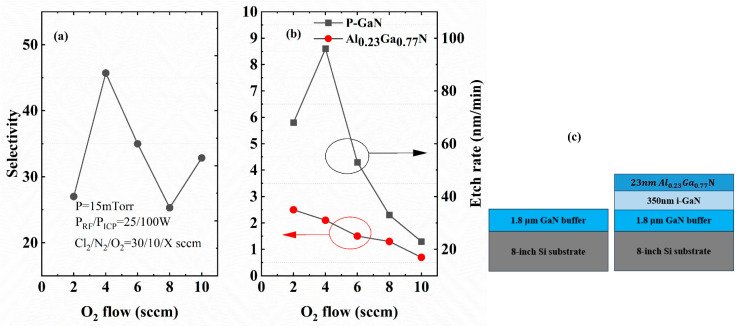
Dependence of (**a**) selectivity between p-GaN and Al_0.23_Ga_0.77_N on O_2_ flow rate and (**b**) the corresponding etch rates. (**c**) Schematic structures of sample A with a 1.8 μm thick GaN buffer layer, and sample B with a 23 nm thick AlGaN barrier layer.

Experiments utilized GaN and Al_0.23_Ga_0.77_N samples grown on Si substrates via MOCVD. To facilitate etch depth measurements, Sample A (GaN) was grown up to the buffer layer termination, whereas Sample B (Al_0.23_Ga_0.77_N) was designed with an intentionally thickened barrier layer without p-GaN capping, as schematically shown in Figure 4b. Substrates were diced into 10 × 10 mm^2^ specimens, where a 200 nm SiO_2_ layer deposited by plasma-enhanced CVD served as an etching mask. Etch depths under varying O_2_ flows (0–10 sccm) were quantified using atomic force microscopy (AFM) to determine the etch rates. Notably, the process demonstrated excellent uniformity and repeatability. Figure 4b systematically compares the etch rates of p-GaN and Al_0.23_Ga_0.77_N layers under varying oxygen flow rates, with calculated selectivity ratios (p-GaN: AlGaN), as shown in Figure 4a, illustrating process tunability.

As shown in Figure 4, the ICP etch rate of Al_0.23_Ga_0.77_N decreases significantly with increasing O_2_ flow, while the p-GaN etch rate remains consistently higher than that of Al_0.23_Ga_0.77_N. This disparity arises from the formation of an etch-resistant (Al,Ga) Ox film on the Al_0.23_Ga_0.77_N surface during oxygen-assisted etching. Notably, the p-GaN etch rate initially increases with O_2_ flow, peaking at 96 nm/min for 4 sccm O_2_, before declining to 20 nm/min at 10 sccm. In contrast, the Al_0.23_Ga_0.77_N etch rate decreases monotonically with rising O_2_ flow, achieving maximum selectivity (p-GaN:AlGaN) at 4 sccm. At 10 sccm O_2_, the process achieves both low etch rates and high selectivity, making it suitable for high etching accuracy applications.

**Figure 5:**
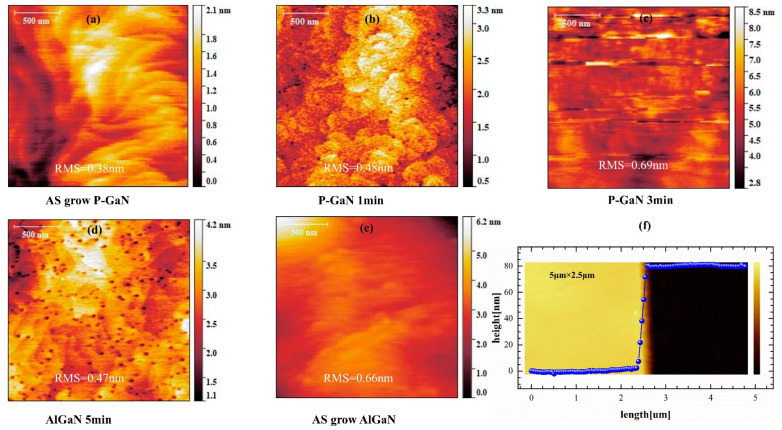
(**a**–**c**) AFM images of p-GaN surfaces subjected to selective etching for 0, 1, and 3 min, respectively. (**d**,**e**) Corresponding AFM images of AlGaN surfaces after 0 and 5 min of selective etching. Root-mean-square (RMS) roughness values are annotated beneath each image. (**f**) Step height profile measured by AFM after 5 min of etching, revealing a step height of ~80 nm. (insert: AFM image of patterned sample after 300 s etching).

For process validation, the full epitaxial stack shown in Figure 1a was diced into four 5 × 5 mm^2^ specimens. Selected regions were protected by a 200 nm-thick SiO_2_ mask deposited via PECVD and patterned through photolithography. Dry etching was subsequently performed using a Cl_2_/O_2_/N_2_ gas mixture (30:10:10 sccm ratio) at 15 mTorr, with ICP source power and bias power maintained at 100 W and 25 W, respectively. Etching durations of 0, 1, 3, and 5 min were applied to the corresponding specimens. After etching, the SiO_2_ mask was removed using BOE (buffered oxide etch), and characterization was performed by AFM. The experimental results are presented in Figure 5.

Figure 5 displays 2 μm × 2 μm AFM images comparing the etch duration effects, including reference images of as-grown p-GaN and Al_0.23_Ga_0.77_N surfaces. The root mean square (RMS) roughness of the p-GaN surface progressively increased from 0.38 nm (unetched) to 0.48 nm after 1 min of etching, further rising to 0.69 nm following 3 min of etching. Following complete p-GaN removal with subsequent 1 min overetching, the exposed Al_0.23_Ga_0.77_N surface showed reduced roughness (0.47 nm), representing an approximately 28% decrease compared to the as-grown reference surface (0.66 nm). This surface quality enhancement confirms the low-damage characteristics inherent to the high-selectivity, low-rate etching process. The residual features observed on the etched Al_0.23_Ga_0.77_N surface (Figure 5d) originate from intrinsic material features associated with the epitaxial growth process.

AFM step-height measurements quantified a total etch depth of ~80 nm, matching the nominal p-GaN layer thickness. Notably, the Al_0.23_Ga_0.77_N barrier layer exhibited less than 1 nm thickness loss during the 1 min overetch phase, demonstrating exceptional selectivity. This controlled barrier modification preserves the 2DEG density in access regions, a critical factor for maintaining low on-resistance in HEMT operation.

### 3.2. Non-Annealed Ohmic Contacts

**Figure 6:**
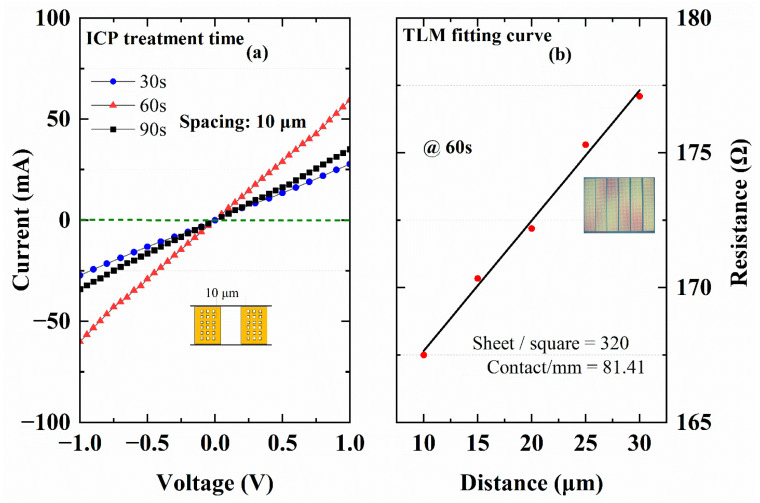
(**a**) IV characteristics of the TLM pattern with different treatment times. (**b**) An ohmic result by fitting the TLM curve, and the inset is the TLM test patterns characterized by optical microscopy, demonstrating well-defined metallic electrodes with a spacing of 10–30 μm.

ICP etching was implemented in ohmic regions to simultaneously fabricate recessed patterns and generate nitrogen vacancies at the contact interface, enabling non-annealed ohmic contact formation. During ICP dry etching, higher bias power improved current linearity, promoting ohmic behavior. Based on previous reports [11], a 100 W bias power was selected to optimize carrier transport. The ICP etching duration was precisely regulated (30–90 s) to achieve optimal contact resistance. Figure 6a shows the current-voltage (I-V) characteristics of recessed ohmic contacts under varying etch conditions, with the highest current density observed at 60 s etching.

Transfer length method (TLM) measurements yielded a contact resistance ($R_{c}$) of 81.41 Ω·mm and a sheet resistance ($R_{sh}$) of 320 Ω/□, higher than the values obtained from conventional annealed contacts (850 °C rapid thermal annealing for 30 s). This elevated resistance primarily stems from surface damage induced by ICP etching and the reduced Al_0.23_Ga_0.77_N barrier layer in p-GaN-capped heterostructures. Figure 6b presents the TLM fitting curve for the 60 s ICP-etched contact, validating the feasibility of implementing this non-annealed process in p-GaN/AlGaN/GaN HEMTs while maintaining acceptable ohmic performance.

The relatively thin barrier layer (13 nm) and low Al composition (23%) in these structures suggest that contact resistance could be further reduced by optimizing epitaxial growth conditions. To simplify fabrication, the current process simultaneously defines ohmic recesses and mesa isolation in a single lithography step. However, the mesa isolation depth requirement (≥120 nm) inevitably limits the minimum ICP etching duration to 30 s. Introducing an additional lithography step to decouple these processes, along with precise tuning of ICP parameters, could enable shorter ohmic-specific etching times and lower $R_{c}$.

### 3.3. Device Performance

**Figure 7:**
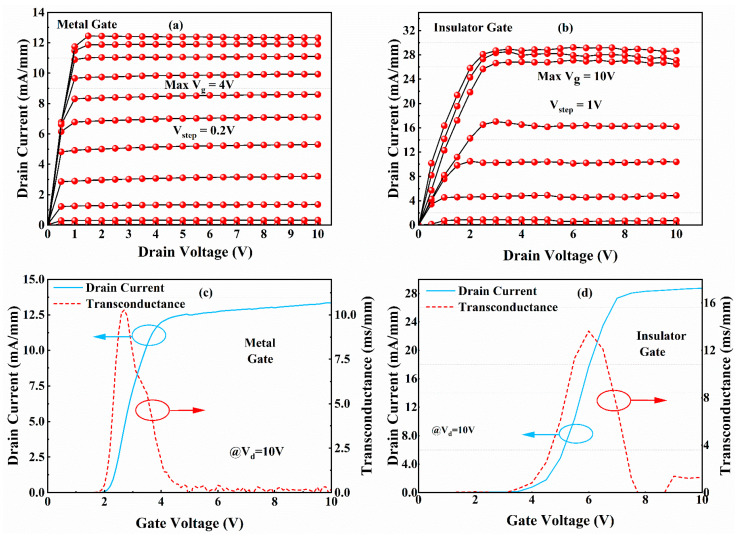
Output characteristics of devices with a metal gate structure (**a**) and an insulator gate structure (**b**). Transfer characteristics and transconductance curves of the metal gate structure (**c**) and the insulator gate structure (**d**).

Devices were fabricated with a gate length $L_{g}$ = 2 μm, an effective gate width $W_{g}$ = 100 μm, a gate-source spacing ($L_{gs}$) of 2 μm, and a gate-drain spacing ($L_{gd}$) of 11 μm, as shown in the optical micrographs of the fabricated devices (Figure 3a). Figure 7a,b compare the output characteristics of insulator gate ($V_{g}$ = −2 to +10 V) and metal gate ($V_{g}$ = −3 to +4 V) devices. The insulated gate device exhibits an extended gate voltage swing compared with the metal gate device. Both devices demonstrate clean pinch-off characteristics at their maximum gate biases (4 V and 10 V, respectively). This improvement originates from the gate-first fabrication sequence—gate metal deposition precedes mesa isolation—which eliminates edge-type leakage pathways typically induced by mesa edge defects in conventional gate-last processes.

The metal gate device exhibited a maximum drain current density ($I_{D}$,max) of 13 mA/mm at $V_{g}$ = 4 V, whereas the insulated gate device demonstrated $I_{D}$,max = 29 mA/mm at $V_{g}$ = 10 V, representing a 123% enhancement despite identical ohmic contacts and epitaxial structures. This performance enhancement was consistently observed in transfer characteristics (Figure 7c). The insulated gate device showed a threshold voltage ($V_{th}$) of 4.5 V (extracted via linear extrapolation), significantly higher than the metal gate device’s $V_{th}$ of 2 V. This positive $V_{th}$ shift originates from voltage division across the ${SiN}_{x}$ gate dielectric, p-GaN layer, and channel region. The extended gate swing to 10 V further confirms effective gate leakage suppression by the ${SiN}_{x}$ dielectric.

Notably, the maximum transconductance (gm,max = 13.6 mS/mm) of the insulated gate device shows no significant improvement over the metal gate version. This limitation arises from the increased gate-to-2DEG distance introduced by the dielectric layer. Device performance can be improved by the substitution of ${SiN}_{x}$ with high-κ dielectrics ALD-based optimization of dielectric thickness and interface quality. The lack of secondary gm peaks (characteristic of hole injection [5]) indicates effective suppression of valence band parasitic conduction even at high gate biases.

**Figure 8:**
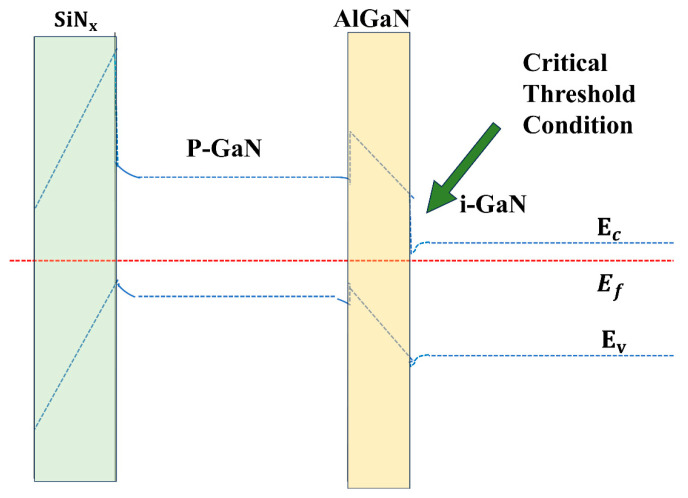
Schematic diagram of energy band at $V_{th}$ for the metal and insulator gate.

Figure 8 illustrates energy band diagrams at threshold conditions for metal gate and insulated gate devices. For the metal gate device, the conduction band minimum ($E_{C}$) achieves alignment with the Fermi level ($E_{F}$) at $V_{th}$, thereby enabling the 2DEG recovery. The insulator gate device requires a higher gate bias to overcome the dielectric barrier while inducing equivalent band bending for 2DEG formation. Given identical epitaxial structures (dielectric excluded), the charge requirement (Q) for 2DEG activation is identical, which leads to the establishment of the following relationship:

(1) $$Q_{Metal\;Gate}=Q_{Insulator\;Gate}$$

(2) $$Q_{Metal\;Gate}=C_{ox}V_{th}(Metal)$$

(3) $$Q_{Insulator\;Gate}=\frac{1}{\frac{1}{C_{SiN}}+\frac{1}{C_{Ox}}}V_{th}(Insulator\;Gate)$$

where $C_{Ox}$ denotes the AlGaN barrier capacitance and $C_{SiN}$ represents the ${SiN}_{x}$ dielectric capacitance. From Equations (1)–(3), the threshold voltage relationship can be derived as follows:

$$V_{th}(Insulator\;Gate)=V_{th}(Metal)\times (1+\frac{C_{Ox}}{C_{SiN}})$$

This establishes an inversely proportional relationship between Vth enhancement and ${SiN}_{x}$ capacitance. Under varying ${SiN}_{x}$ thicknesses, the electric field ($E_{SiN}$) remains constant throughout the heterostructure: $U_{SiN}$ = $E_{SiN}$ × $t_{SiN}$. Here, $U_{SiN}$ denotes the voltage drop across the dielectric, $E_{SiN}$ represents the electric field, and $t_{SiN}$ corresponds to the dielectric thickness. Consequently, $V_{th}$ (insulator gate) exhibits direct proportionality to $t_{SiN}$, since thicker dielectrics require higher gate biases to maintain equivalent E-field conditions.

**Figure 9:**
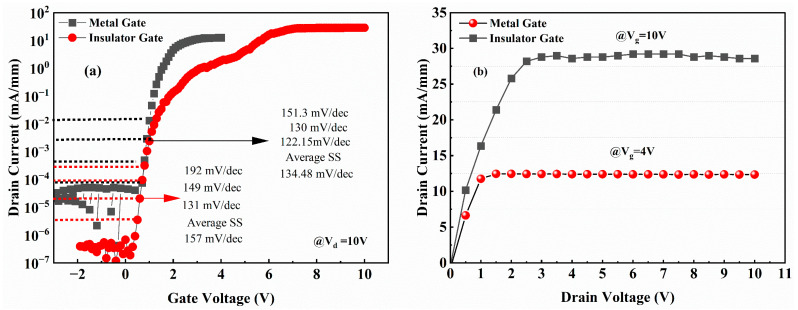
(**a**) Semi-logarithmic transfer characteristics of metal gate and insulated gate devices, with extracted average subthreshold swing (SS) values of 134.48 mV/dec and 157 mV/dec, respectively. (**b**) Output characteristics measured at gate biases of VGS = 4 V (metal gate) and 10 V (insulator gate).

Figure 9a compares semi-log transfer characteristics, revealing an enhanced on/off ratio of ~$10^{7}$ for the insulator gate device—two orders of magnitude higher than the metal gate device. The insulator gate exhibits superior gate leakage suppression, demonstrating significantly lower gate currents than those of metal gates. Subthreshold swing (SS) analysis yields average values of 157 mV/dec (insulated-gate) and 134.48 mV/dec (metal-gate) at $V_{d}$ = 10 V. Notably, both devices lack passivation layers, yet the metal gate SS outperforms the reported values in [20], highlighting the intrinsic interface quality benefits achieved through the gate-first process.

The on-resistance (Ron) was extracted from the linear region of the output characteristics at $V_{g}$ = 4 V (metal-gate) and $V_{g}$ = 10 V (insulated-gate), as documented in Figure 9b. The insulated gate device exhibits Ron = 347 Ω, higher than the metal gate counterpart (321 Ω); it is important to emphasize that both ohmic contact resistance and access region resistance values remain comparable between the two device configurations. This minimal difference (<8%) confirms comparable channel resistance and indicates no significant degradation in 2DEG carrier concentration or mobility due to the ${SiN}_{x}$ dielectric integration.

Further improvements in leakage and switching performance could be achieved through passivation layer optimization and tailored dielectric stress engineering. The absence of passivation in this work provides fundamental insights for the subsequent process optimization.

## 4. Conclusions

In conclusion, we have presented a gate-first self-aligned fabrication scheme for p-GaN HEMTs, which integrates high-selectivity dry etching, minimal etch damage, and non-annealed ohmic contacts. The Cl_2_/O_2_/N_2_-based ICP-RIE process developed achieves high selectivity in etching p-GaN, ensuring that the AlGaN barrier layer remains intact while preserving the key two-dimensional electron gas (2DEG) density. The integrated non-annealed ohmic process eliminates the need for high-temperature annealing, significantly simplifying the manufacturing process. Notably, the non-annealed ohmic contact process results in a contact resistance of 81.41 Ω·mm, demonstrating effective ohmic contact formation without the risk of gate degradation associated with high-temperature annealing in conventional gate-first methods.

From a manufacturing perspective, the gate-first process offers significant advantages in terms of cost and simplicity. By integrating the gate metal as both etch mask and electrical contact while eliminating additional photolithography and etching steps, the approach reduces complexity while minimizing potential alignment errors. Furthermore, the process requires fewer steps, avoids high-temperature treatments, and provides a cost-effective solution for rapid device fabrication and performance testing.

A normally off AlGaN/GaN heterojunction field-effect transistor with SiNx gate die-electric was fabricated using the proposed gate-first process. The device exhibited excellent electrical performance with a threshold voltage shift of +4.5 V, an on/off current ratio exceeding $10^{7}$, a gate voltage swing of 10 V, and outstanding pinch-off characteristics. The dielectric integration further enhances the stability and performance of the device due to the expanded gate voltage swing and superior gate leakage suppression.

Overall, the proposed fabrication method not only provides high-performance, normally off GaN-based power devices, but also simplifies the manufacturing process, making it a promising solution for cost-effective, high-quality semiconductor device production.

## Data Availability

The original contributions presented in this study are included in the article. Further inquiries can be directed to the corresponding author.
